# Trial of labour after caesarean section and the risk of neonatal and infant death: a nationwide cohort study

**DOI:** 10.1186/s12884-017-1255-2

**Published:** 2017-02-27

**Authors:** Sinéad M. O’Neill, Esben Agerbo, Ali S. Khashan, Patricia M. Kearney, Tine Brink Henriksen, Richard A. Greene, Louise C. Kenny

**Affiliations:** 1 The Irish Centre for Fetal and Neonatal Translational Research (INFANT), Cork University Maternity Hospital, University College Cork, Wilton, Cork, Ireland; 2 National Perinatal Epidemiology Centre (NPEC), Department of Obstetrics and Gynaecology, Cork University Maternity Hospital, 5th Floor Wilton, Cork, Ireland; 3 Centre for Integrated Register-based Research (CIRRAU), National Centre for Register-based Research (NCRR), Aarhus University, Aarhus, Denmark; 4 Department of Epidemiology and Public Health, University College Cork, Western Gateway Building, Cork, Ireland; 5 Perinatal Epidemiology Research Unit, Department of Paediatrics, Aarhus University Hospital, Skejby, Aarhus N, DK-8200 Denmark

**Keywords:** Trial of labour, Neonatal death, Repeat Caesarean section

## Abstract

**Background:**

Caesarean section (CS) rates are increasing worldwide and as a result repeat CS is common. The optimal mode of delivery in women with one previous CS is widely debated and the risks to the infant are understudied. The aim of the current study was to evaluate if women with a trial of labour after caesarean (TOLAC) had an increased odds of neonatal and infant death compared to women with an elective repeat CS (ERCS).

**Methods:**

A population register-based cohort study was conducted in Denmark between 1982 and 2010. All women with two deliveries [in which the first was a CS, and the second was an uncomplicated, term delivery (*n* = 61,626)] were included in the study. Logistic regression models were used to report adjusted odds ratios (AOR) and 95% confidence intervals (CI) of the odds of death according to mode of delivery. The main outcome measures were neonatal death (early and late) and infant death.

**Results:**

Women with a TOLAC had an increased odds of neonatal death (AOR 1 · 87, 95% CI 1 · 12 to 3 · 12) due to an increased risk of early neonatal death (AOR 2 · 06, 95% CI 1 · 19 to 3 · 56) and no effect on late neonatal death (AOR 0 · 97, 95% CI 0 · 22 to 4 · 32), or infant death (AOR 1 · 12, 95% CI 0 · 79 to 1 · 59) when compared to the reference group of women with an ERCS. There was evidence of a cohort effect as the increased odds of neonatal death (AOR 3 · 89, 95% CI 1 · 33 to 11 · 39) was most significant in the earlier years (1982–1991) and gradually disappeared (AOR 1 · 01, 95% CI 0 · 44 to 2 · 31) in the later years (2002–2010).

**Conclusions:**

Although an increased risk of neonatal death was found in women with a TOLAC, there was evidence of a cohort effect, which showed this increased odds disappearing over time. Advances in modern healthcare including improved monitoring and earlier detection of underlying pregnancy complications may explain the findings.

**Electronic supplementary material:**

The online version of this article (doi:10.1186/s12884-017-1255-2) contains supplementary material, which is available to authorized users.

## Background

Caesarean section (CS) rates are increasing worldwide, and currently CS is the most commonly performed surgical procedure in women of childbearing age in the United States (US) [1]. Proposed driving forces include increasing maternal age at first pregnancy [2], increasing body mass index (BMI) [3], elective CS for breech presentation and more recently maternal request for a CS [4, 5], as well elective repeat CS (in women with a first CS) [ERCS] [6]. Trial of labour after Caesarean section (TOLAC) rates have decreased significantly in some countries [7], largely due a reported increased risk of uterine rupture and perinatal asphyxia in women undergoing a TOLAC compared to a planned ERCS [8–11], whilst remaining unchanged in others [12, 13]. Rising CS rates have generated much debate on the benefits and risks to the mother and her offspring attributable to mode of delivery, particularly whether or not to attempt a TOLAC in the subsequent delivery [14]. Numerous studies have focused on maternal outcomes reporting an increased risk of uterine rupture, placenta accreta and placenta previa [15–17]. Evidence remains to be explored regarding infant outcomes in the subsequent pregnancy following first CS delivery particularly early and late neonatal death [18, 19]. Controversy remains on whether a TOLAC or an ERCS is preferable for women with one prior CS. In the absence of clinical trials on the topic which are difficult to conduct, population register-based research incorporating detailed obstetric information remains the optimum study design to address this important question.

The objective of the current study was to examine the odds of neonatal death, early and late neonatal death and infant mortality in a cohort of women with an uncomplicated term delivery who had a TOLAC or ERCS following a first CS.

## Methods

### Study design, data source and population studied

**Fig. 1:**
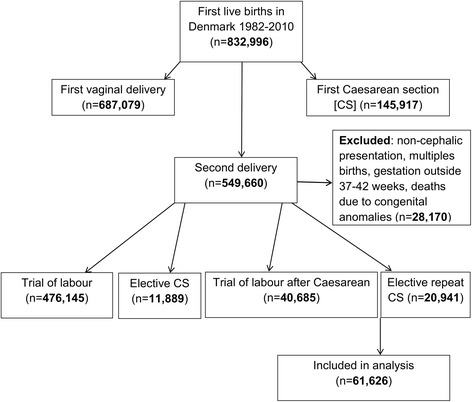
Flowchart of the study population

A cohort study design using the Danish Civil Registration System (CRS) data was used to identify all women with their first two deliveries in Denmark between January 1^st^ 1982 and December 31^st^ 2010 (of which the first was a CS, and the second an ERCS or TOLAC). In order to be eligible for inclusion in this study the second delivery had to be an uncomplicated, term delivery (i.e. a singleton, cephalic delivery between 37 and 42 weeks’ gestation). After removal of ineligible women and deaths due to congenital anomalies, there were 61,626 women eligible for inclusion in the analyses (Fig. 1).

The Danish CRS was established in 1968 originally for administrative purposes but has since become an invaluable source of population-based data for use in epidemiological research [20, 21]. The CRS uses a unique identifier known as the civil person register (CPR) which enables researchers to link data from different registers as well as the continuous follow-up of individuals living in Denmark. For the current study data from the CRS were linked to the Danish Medical Birth Registry (MBR) [22, 23], the National Hospital Register (NHR) [24], the Danish Causes of Death Register [25] and socioeconomic data were obtained from *Statistics Denmark* [26] using the unique CPR number.

### Exposure

Mode of delivery was categorised as follows: 1) first CS and subsequent ERCS [reference group] (*n* = 20,941), 2) first CS and subsequent TOLAC (*n* = 40,685). For the purposes of this study the TOLAC group included any patient with a previous CS who did not have an ERCS. It therefore includes patients who have a vaginal birth after Caesarean (VBAC, i.e. a successful TOLAC) or an emergency CS (which may have arisen from a failed TOLAC or may have been done for women with a planned CS who attends in labour, with ruptured membranes not in labour, etc. and had an ‘emergency/semi-emergency’ CS). The groups represent what happens in a real life situation, i.e. ERCS (planned) versus all other modes of delivery.

Mode of delivery recorded in the Danish registers is the ‘actual’ mode of delivery (which may differ from the woman’s intended mode of delivery). For example, a woman may attempt a TOLAC and fail, ending up with an emergency CS delivery. In the Danish registries, an elective CS is defined as a pre-planned (before initiation of labour) procedure whilst an emergency CS is unplanned. In this study, we only have information on the final ‘actual’ mode of delivery recorded in the registry using the appropriate codes (elective CS *DO820*; emergency CS *DO821*).

### Main outcome measures

The primary outcome was neonatal death: death of a live new born within ≤28 days. Secondary outcomes included: 1) Early neonatal death: death of a live new born within ≤7 days; 2) Late neonatal death: death of a live new born > 7 days ≤ 28 days and 3) Infant death: the death of a child within ≤365 days.

### Statistical analyses

Crude and adjusted logistic regression models were computed to estimate the odds of each outcome in women with a TOLAC compared to women with an ERCS using crude odds ratios (ORs) or adjusted ORs (AORs) and 95% confidence intervals (CIs). Adjusted models were *a priori* defined as follows:

Model 1: key potential confounders available for the entire study period from the second live birth including maternal age, country of origin, educational attainment, mother and father’s gross income, marital status, infant birthplace, infant birth weight, birth year and history of pregnancy loss (1982–2010, cohort *n* = 61,626).

### Testing for a cohort effect

In order to test for the cohort effect and temporal changes over time, the cohort was split into three different time periods (1982–1991, 1992–2001, 2002–2010) and the analyses repeated.

### Subgroup and sensitivity analyses

A subgroup analysis restricted to women delivering between 38 and 40 weeks’ gestation was conducted to investigate the effect of gestational age on the outcomes of interest in overdue or induced pregnancies with a TOLAC. In addition, data for smoking, co-morbidities and BMI were only available for specific time periods. Sensitivity analyses were conducted restricting the cohort to the specific time periods for which data were available on smoking, co-morbidities and BMI (with and without adjusting for these covariates).

- Model 1: adjusted for key potential confounders available for the entire study period including maternal age, country of origin, educational attainment, mother and father’s gross income, marital status, infant birthplace, infant birth weight, birth year and history of pregnancy loss plus smoking status (1991–2010, cohort *n* = 50,880).
- Model 2: adjusted for Model 1 plus comorbidities in the second live birth including hypertension, eclampsia, preeclampsia, fetal distress and gestational diabetes (1994–2010, cohort *n* = 45,979).
- Model 3: adjusted for Model 2 plus BMI (2004–2010, cohort *n* = 22,672).

Where a variable had missing data, the variable was re-coded to include missing data as a separate category (for example, for maternal smoking, 1 = smoker, 2 = non-smoker, 3 = missing) and included in the various analyses. As outlined by Vach and Blettner, adding missing data as a separate category where the proportion of missing data is small (as in this study) should not impact greatly on the effect estimates [27]. All analyses were conducted using SAS© version 9.4 software (SAS Institute, Inc., Cary, NC, USA) and the PROC LOGISTIC [28] command. Approval to use the data was obtained from the Danish National Board of Health and *Statistics Denmark* for the current study.

## Results

### Characteristics of the study population

The study included 61,626 women with at least two live births. These women were subdivided into 40,685 women with a TOLAC (66%), and 20,941 (34%) women with an ERCS, (Fig. 1). Maternal characteristics of the study group are outlined in [Table 1] and infant characteristics are outlined in [Table 2].

**Table 1:** Maternal characteristics in the second live birth in Denmark, 1982–2010

	First CS		
	Elective repeat CS	Trial of labour after CS	Total
Maternal characteristics of the second delivery			
Age in years, <20	24 (0%)	133 (0%)	157 (0%)
20–25	1,920 (9%)	5,655 (14%)	7,575 (12%)
26–30	6,997 (33%)	16,582 (41%)	23,579 (38%)
31–35	8,096 (39%)	14,349 (35%)	22,445 (36%)
36–40	3,423 (16%)	3,660 (9%)	7,083 (11%)
41+	481 (2%)	306 (1%)	787 (1%)
Origin, Denmark	18,685 (89%)	36,757 (90%)	55,442 (90%)
Other	2,183 (10%)	3,834 (9%)	6,017 (10%)
Unknown	73 (0%)	94 (0%)	167 (0%)
Marital status, Married	7,514 (36%)	13,567 (33%)	21,081 (34%)
Divorced/separated/widowed	573 (3%)	748 (2%)	1,321 (2%)
Co-habiting	12,633 (60%)	25,917 (64%)	38,550 (63%)
Unknown	221 (1%)	453 (1%)	674 (1%)
Educational attainment, Primary	4,496 (21%)	9,817 (24%)	14,313 (23%)
High school	10,193 (49%)	20,057 (49%)	30,250 (49%)
Third level degree	4,183 (20%)	7,432 (18%)	11,615 (19%)
Masters/PhD	1,447 (7%)	2,183 (5%)	3,630 (6%)
Unknown	622 (3%)	1,196 (3%)	1,818 (3%)
Mother’s gross income (quartiles), 25	2,997 (14%)	5,766 (14%)	8,763 (14%)
50	3,954 (19%)	7,789 (19%)	11,743 (19%)
75	9,513 (45%)	19,299 (47%)	28,812 (47%)
100	4,339 (21%)	7,514 (18%)	11,853 (19%)
Unknown	138 (1%)	317 (1%)	455 (1%)
^a^Smoker, No	14,387 (82%)	25,814 (78%)	40,201 (79%)
Yes	2,665 (15%)	6,733 (20%)	9,398 (18%)
Unknown	566 (3%)	715 (2%)	1,281 (3%)
Characteristic of the second delivery			
Father’s gross income (quartiles), 25	1,497 (7%)	3,156 (8%)	4,653 (8%)
50	2,264 (11%)	4,765 (12%)	7,029 (11%)
75	6,266 (30%)	12,702 (31%)	18,968 (31%)
100	10,582 (51%)	19,412 (48%)	29,994 (49%)
Unknown	332 (2%)	650 (2%)	982 (2%)
^b^BMI (kg/m^2^) <18.5	523 (5%)	638 (5%)	1,161 (5%)
18.5–25	5,849 (55%)	7,412 (62%)	13,261 (58%)
26–30	2,198 (21%)	2,092 (18%)	4,290 (19%)
31–35	1,064 (10%)	753 (6%)	1,817 (8%)
36+	643 (6%)	347 (3%)	990 (4%)
Unknown	439 (4%)	714 (6%)	1,153 (5%)
^c^Preeclampsia, eclampsia	342 (2%)	613 (2%)	955 (2%)
^d^Fetal distress	98 (1%)	6,358 (22%)	6,456 (14%)
^e^Gestational diabetes	645 (4%)	504 (2%)	1,149 (3%)
^f^Hypertension	203 (1%)	337 (1%)	540 (1%)
Previous stillbirth	494 (2%)	371 (1%)	865 (1%)
Previous miscarriage	3,288 (16%)	6,675 (16%)	9,963 (16%)
Previous ectopic pregnancy	863 (4%)	1,681 (4%)	2,544 (4%)

Data are n (%).^a^Smoking data available from 1991 to 2010 (cohort *n* = 50,880). ^b^BMI: Body mass index, data available from 2004 to 2010 only (cohort *n* = 22,672). ^c, d, e, f,^ Preeclampsia, eclampsia; fetal distress; gestational diabetes, hypertension: data available from 1994 to 2010 (cohort *n* = 45,979). *CS* Caesarean section

**Table 2:** Infant characteristics in the second live birth in Denmark, 1982–2010

Infant characteristics of the second delivery	First CS	
	Elective repeat CS	Trial of labour after CS
Infant sex, male	10,845 (52%)	20,472 (50%)
Female	10,096 (48%)	20,213 (50%)
Birthplace, Capital (Copenhagen)	3,809 (18%)	6,352 (16%)
Capital suburbs	2,224 (11%)	5,409 (13%)
Provincial city	2,641 (13%)	4,852 (12%)
Provincial town	5,846 (28%)	11,184 (27%)
Rural area	6,369 (30%)	12,807 (31%)
Unknown	52 (0%)	81 (0%)
Apgar score <7 at 5 min	190 (1%)	1,016 (3%)
Birth weight (g), <2,500	1,440 (7%)	6,165 (15%)
2500–3500	8,179 (39%)	18,805 (46%)
3500–4500	9,556 (46%)	14,358 (35%)
4500+	1,569 (7%)	1,129 (3%)
Unknown	197 (1%)	228 (1%)
Gestational age, weeks, mean ± standard deviation	39.06 (1.2)	40.28 (1.3)
Admission to NICU^a^	645 (5%)	414 (3%)

Data are n (%) or mean ± standard deviation unless otherwise stated. *CS* Caesarean section, *NICU* Neonatal intensive care unit. ^a^Admission to NICU: data available from 2002 to 2010 (*n* = 27,868)

The rate of successful TOLAC (VBAC) did not vary significantly over the entire study period ranging from 5 to 8% (see Additional file 1).

### Neonatal death, early neonatal death, late neonatal death and infant death

Neonatal death (≤28 days): An increased odds of neonatal death was found in women with a TOLAC (AOR 1 · 87, 95% CI 1 · 12, 3.12) compared to the reference group of women with an ERCS. Early neonatal death (≤7 days after birth): Compared to the reference group of women with an ERCS, women with a TOLAC had more than twice the odds of early neonatal death (AOR 2 · 06, 95% CI 1 · 19, 3 · 56), (see Table 3).

**Table 3:** Neonatal death and infant death according to mode of delivery in a Danish cohort, 1982–2010

Mode of delivery 1^st^ and 2^nd^ birth (Number of events)	Neonatal death (≤28 days) *n* = 95	
	OR (95% CI)	^a^Model 1 AOR (95% CI)
CS – ERCS (*n* = 21)	Ref	Ref
CS – TOLAC (*n* = 74)	1.82 (1.12, 2.95)	1.87 (1.12, 3.12)
Mode of delivery 1^st^ and 2^nd^ birth (Number of events)	Early neonatal death (≤7 days) *n* = 86	
	OR (95% CI)	^a^Model 1 AOR (95% CI)
CS – ERCS (*n* = 18)	Ref	Ref
CS – TOLAC (*n* = 68)	1.95 (1.16, 3.27)	2.06 (1.19, 3.56)
Mode of delivery 1^st^ and 2^nd^ birth (Number of events)	Late neonatal death (>7 days, ≤28 days) *n* = 9	
	OR (95% CI)	^a^Model 1 AOR (95% CI)
CS – ERCS (*n* = 3)	Ref	Ref
CS – TOLAC (*n* = 6)	1.03 (0.26, 4.12)	0.97 (0.22, 4.32)
Mode of delivery 1^st^ and 2^nd^ birth (Number of events)	Infant death (≤365 days) *n* = 171	
	OR (95% CI)	^a^Model 1 AOR (95% CI)
CS – ERCS (*n* = 49)	Ref	Ref
CS – TOLAC (*n* = 122)	1.28 (0.92, 1.79)	1.12 (0.79, 1.59)

Data are crude and adjusted odds ratios with 95% confidence intervals. *OR* odds ratio, *AOR* adjusted odds ratio, *CI* confidence interval, *ERCS* Elective repeat caesarean section, *TOLAC* Trial of labour after caesarean section

^a^Model 1: adjusted for key covariates in the second birth including maternal age, maternal country of origin, educational attainment, mother and father’s gross income, marital status, infant birthplace and infant birth weight, history of pregnancy loss and birth year (cohort *n* = 61,626)

Late neonatal death (>7 days, ≤ 28 days after birth): Compared to the reference group, women with a TOLAC had no increased odds of late neonatal death (AOR 0 · 97, 95% CI 0 · 22, 4 · 32). Infant death (≤365 days after birth): There was no association (AOR 1.12, 95% CI 0.79, 1.59) between women with a TOLAC and infant death compared to women with an ERCS (see Table 3).

### Testing for a cohort effect

To test for the cohort effect, the cohort was split into three time periods. The increased odds of neonatal death among women with a TOLAC was greatest in the earliest years (1982–1991) with an AOR of 3 · 89 (95% CI 1 · 33, 11 · 39) compared to women with an ERCS (see Table 4).

**Table 4:** Neonatal death and infant death according to mode of delivery by time period (cohort effect)

Mode of delivery 1^st^ and 2^nd^ births	Neonatal death (≤28 days) *n* = 95 in entire cohort 1982–2010		
	^a^Model 1 AOR (95% CI) (35 deaths)	^b^Model 2 AOR (95% CI) (34 deaths)	^c^Model 3 AOR (95% CI) (26 deaths)
CS – ERCS	Ref	Ref	Ref
CS – TOLAC	3.89 (1.33, 11.39)	2.87 (0.85, 9.70)	1.01 (0.44, 2.31)
Mode of delivery 1^st^ and 2^nd^ birth	Infant death (≤365 days) *n* = 171 in entire cohort 1982–2010		
	^a^Model 1 AOR (95% CI) (69 deaths)	^b^Model 2 AOR (95% CI) (64 deaths)	^c^Model 3 AOR (95% CI) (38 deaths)
CS – ERCS	Ref	Ref	Ref
CS – TOLAC	1.24 (0.70, 2.21)	1.37 (0.70, 2.69)	0.81 (0.42, 1.59)

Data are adjusted odds ratios with 95% confidence intervals. *AOR* adjusted odds ratio, *CI* confidence interval, *ERCS* Elective repeat caesarean section, *TOLAC* Trial of labour after caesarean

^a^Model 1: Cohort restricted to first time period (1982–1991, cohort *n* = 11,698) and adjusted for key covariates in the second birth including maternal age, maternal country of origin, educational attainment, mother and father’s gross income, marital status, infant birthplace and infant birth weight, history of pregnancy loss and birth year

^b^Model 2^:^ Cohort restricted to second time period (1992–2001, cohort *n* = 22,060) and adjusted for key covariates as in Model 1

^c^Model 3: Cohort restricted to last time period (2002–2010, cohort *n* = 27,868) and adjusted for key covariates as in Model 1

**Fig. 2:**
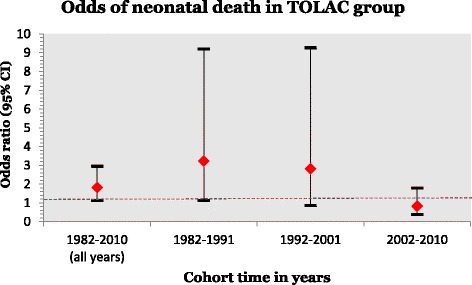
Odds ratio of neonatal death in the trial of labour after caesarean section (TOLAC) group

This decreased over time to an AOR of 2 · 87 (95% CI 0 · 85, 9 · 70) between 1992 and 2001, and disappeared in the most recent years of 2002–2010 (AOR 1 · 01, 95% CI 0 · 44, 2 · 31). This decreasing trend is displayed graphically (see Fig. 2).

### Subgroup and sensitivity analyses

A subgroup analysis restricted to include women delivering between 38 and 40 weeks was conducted to assess the effect of gestational age on overdue or induced women with a TOLAC compared to the reference group of women with an ERCS (see Additional file 2). Women with a TOLAC had an increased odds of neonatal death (AOR 1 · 78, 95% CI 1 · 03, 3 · 08) and early neonatal death (AOR 1 · 99, 95% CI 1 · 11, 3 · 58). No association was found for late neonatal death (AOR 0 · 71, 95% CI 0 · 13, 3 · 76) or infant death (AOR 1 · 11, 95% CI 0 · 76, 1 · 63) in women with a TOLAC compared to women with an ERCS (see Additional file 2). Further sensitivity analyses were conducted restricting the cohort to the specific time period for which certain covariates were available (smoking, co-morbidities, BMI) but without adjusting for them specifically. For example, smoking data were available from 1991 to 2010 and the cohort was restricted to this time period without adjustment for the effects of smoking. The increased risk of neonatal death overall disappeared in these additional analyses, but did not explain the overall findings (see Additional files 3 and 4).

## Discussion

### Main findings

In the present study the odds of delivery-related neonatal and infant death were assessed in a large cohort of more than 61,000 women. We found evidence of a cohort effect over time, where in the earliest years the increased odds of neonatal death were greatest (over three times higher than that of the women with an ERCS), gradually disappearing over time. Post-hoc analyses summarising the case-mix of TOLAC women according to cohort time were conducted (see Additional file 5). Overall, there was no change in the percentage of women having a TOLAC across the study period as well as in the characteristics of these women including age, educational attainment, gross income and origin. Advances in obstetrics and changes in practice, as well as perhaps changes in the women having a TOLAC are some of the driving forces behind this finding, meaning that the neonatal death rate is now not significantly different with TOLAC, making it a reasonable choice for pregnant women. We found no increased odds of late neonatal death or infant death. It must be acknowledged however that there were only six late neonatal deaths in women with a TOLAC. The association disappeared in additional adjusted analyses accounting for smoking, co-morbidities and BMI, suggesting that generally safety is improving and that better case selection is vital and could make TOLAC even safer. When the cohort was restricted to include women with term deliveries (38–40 weeks gestation), the findings remained the same.

The improved safety in terms of outcomes following TOLAC could be for a multitude of reasons including improvements in CS as an abdominal surgery (for example changes in uterine incision which came into effect in Denmark) and advances in the diagnosis and management of pregnancy and pregnancy-related co-morbidities. Confounding by indication for underlying medical conditions may also explain the increased odds of early neonatal death found in women with a TOLAC due to acute events during delivery for example.

### Strengths and limitations

Our study used population-based registry data which are regularly updated and validated for epidemiological research. The unique CPR identifier enables accurate linkage between the various registers and as a result detailed obstetric data were available for the current study allowing us to adjust for a large number of clinical and demographic potential confounders. The cohort included more than 61,000 women and spanned almost three decades. Therefore, we were also able to test for evidence of a cohort effect over time. We focused on infant mortality as most of the research to date has prioritised outcomes for mothers.

Limitations to this study and the majority of studies conducted prior to this include that we only have data on the actual mode of delivery recorded rather than the ‘planned’ mode of delivery. Although ‘maternal request for a CS’ has been recorded in the Danish registry data since 2002, the number of neonatal deaths was too few in this subgroup to assess whether any association exists in the data. Clinical trials will likely not be forthcoming as women will decline to be randomised according to mode of delivery. Therefore, large-scale observational studies like the current study detailed here remain the best way to study the effects of mode of delivery on subsequent pregnancy outcomes. In addition, we did not have access to data on type of stillbirth (antepartum or intrapartum). This calls for more delicate registrations of events and series of events, indications and procedures, etc. related to reproduction, pregnancy and delivery to enable more detailed analyses of potential associations between e.g. procedures such as CS and adverse events. The study was also carried out using registry data that contain no specific information on the clinical indication for CS. We tried to reduce this problem by adjusting for a number of medical conditions but this could not cover all possible indications. In addition to this, data were only available for certain covariates for specific time periods as outlined earlier. Unmeasured confounders are also a limitation of this study and all observational studies.

### Interpretation

Our findings are in contrast to a previous study conducted in Scotland [29] which found that women with a first CS undergoing a TOLAC had 11 times the odds of delivery-related perinatal death (AOR 11 · 7, 95% CI 1 · 4–101 · 6) compared to women with an ERCS. The confidence intervals reported in the Scottish study are very broad and must be interpreted with caution. Furthermore, the Scottish study included intrapartum stillbirths and did not adjust for medical conditions in addition to BMI as was the case in the current study. The first and only nested randomised control trial (RCT) to date which divided women according to patient preference (*n* = 2,323) or randomisation (*n* = 22) to planned TOLAC or planned ERCS found that among women with one prior CS, planned ERCS compared with planned TOL was associated with a lower risk of fetal death and infant death or serious infant outcome (relative risk [RR] 0 · 39, 95% CI 0 · 19–0 · 80) [30]. Although strengthened by the process of randomisation, very few women actually consented to randomisation.

Ideally further research into the effect of mode of delivery on subsequent pregnancy outcome should incorporate a woman’s ‘intended’ mode of delivery. This is the best way to assess any risks as one would know whether or not a woman was truly eligible to attempt a TOLAC. RCTs would be the optimal method of answering this question however they are difficult to conduct as it is hard to randomise a woman according to mode of delivery and unfeasible for rare outcomes such as perinatal death. Currently however, large population-based data such as the Danish registry data used in this study are the best methods for assessing the effects of mode of delivery and there is a continuous call for improvements in the quality and amount of data collected.

## Conclusions

There are benefits and harms associated with each mode of delivery and the evidence for this is drawn from observational (non-randomised) research studies that may be prone to bias. Any results and conclusions must be interpreted with caution although large-scale population-based studies including the present one offer the best method of estimating the risks in women with one prior CS in the absence of RCTs. Our data provide essential, up to date information for expectant parents as well as healthcare workers to make a more informed decision regarding TOLAC.

## Additional files

Additional file 1: Trial of Labour after Caesarean Section (TOLAC) Rates in Denmark: 1983–2010. (DOCX 16 kb)
Additional file 2: Neonatal and infant death according to mode of delivery – subgroup analysis restricted to women delivering between 38 and 40 weeks gestation. (DOCX 15 kb)
Additional file 3: Neonatal and infant death according to mode of delivery – sensitivity analyses with adjustment for smoking, co-morbidities and body mass index. (DOCX 16 kb)
Additional file 4: Neonatal and infant death according to mode of delivery – sensitivity analyses without adjustment for smoking, co-morbidities and body mass index. (DOCX 16 kb)
Additional file 5: Post-hoc analyses investigating characteristics of women with a TOLAC in Denmark according to cohort time (DOCX 12 kb)

## Abbreviations

AOR: Adjusted Odds Ratio
BMI: Body Mass Index
CI: Confidence Interval
CPR: Central Person Number
CRS: Civil Registration System
CS: Caesarean Section
ERCS: Elective Repeat Caesarean Section
MBR: Medical Birth Register
NHR: National Hospital Register
NICU: Neonatal Intensive Care Unit
OR: Odds Ratio
RCT: Randomised Control Trial
TOL: Trial Of Labour
TOLAC: Trial Of Labour After Caesarean
US: United States
