# Supplementary material for: Trial of labour after caesarean section and the risk of neonatal and infant death: a nationwide cohort study
Source: BMC Pregnancy Childbirth. 2017 Feb 27;17:74. doi: 10.1186/s12884-017-1255-2 (PMC5327578; doi:10.1186/s12884-017-1255-2)
Supplement: Additional file 1: — Trial of Labour after Caesarean Section (TOLAC) Rates in Denmark: 1983–2010. (DOCX 16 kb) [file 12884_2017_1255_MOESM1_ESM.docx]

**Additional File 1 Successful TOLAC (VBAC) Rates in Denmark: 1983-2010**

| **Year** | **Successful TOLAC (VBAC) Rate (%)** | **Change from previous year** |
| --- | --- | --- |
| 1983 | 5.1% | - |
| 1984 | 5.3% | + 0.2% |
| 1985 | 5.8% | + 0.5% |
| 1986 | 6.2% | + 0.4% |
| 1987 | 6.8% | + 0.6% |
| 1988 | 6.8% | - |
| 1989 | 7.3% | + 0.5% |
| 1990 | 7.4% | + 0.1% |
| 1991 | 8.0% | + 0.6% |
| 1992 | 7.6% | - 0.4% |
| 1993 | 7.7% | + 0.1% |
| 1994 | 7.4% | - 0.3% |
| 1995 | 8.0% | + 0.6% |
| 1996 | 7.9% | - 0.1% |
| 1997 | 7.9% | - |
| 1998 | 7.4% | - 0.5% |
| 1999 | 7.7% | + 0.3% |
| 2000 | 7.8% | + 0.1% |
| 2001 | 6.9% | - 0. 9% |
| 2002 | 7.1% | + 0.2% |
| 2003 | 6.6% | - 0.5% |
| 2004 | 7.0% | + 0.3% |
| 2005 | 7.7% | + 0.7% |
| 2006 | 7.9% | + 0.2% |
| 2007 | 7.2% | - 0.7% |
| 2008 | 7.5% | + 0.3% |
| 2009 | 8.0% | + 0.5% |
| 2010 | 7.7% | - 0.3% |

**TOLAC:** Trial of Labour After Caesarean**; VBAC:** Vaginal birth after Caesarean
