# Supplementary material for: Trial of labour after caesarean section and the risk of neonatal and infant death: a nationwide cohort study
Source: BMC Pregnancy Childbirth. 2017 Feb 27;17:74. doi: 10.1186/s12884-017-1255-2 (PMC5327578; doi:10.1186/s12884-017-1255-2)
Supplement: Additional file 2: — Neonatal and infant death according to mode of delivery – subgroup analysis restricted to women delivering between 38 and 40 weeks gestation. (DOCX 15 kb) [file 12884_2017_1255_MOESM2_ESM.docx]

**Additional File 2 Neonatal and infant death according to mode of delivery – subgroup analysis restricted to women delivering between 38 and 40 weeks gestation**

| **Mode of delivery 1^st^ and 2^nd^ birth** | **Neonatal death** (≤28 days) n=74 |
| --- | --- |
|  | **^*^ Model 1 AOR (95% CI)** |
| CS – ERCS | *Ref* |
| CS – TOLAC | 1.78 (1.03, 3.08) |
| **Mode of delivery 1^st^ and 2^nd^ birth** | **Early neonatal death** (≤7 days) n=67 |
|  | **^*^ Model 1 AOR (95% CI)** |
| CS – ERCS | *Ref* |
| CS – TOLAC | 1.99 (1.11, 3.58) |
| **Mode of delivery 1^st^ and 2^nd^ birth** | **Late neonatal death** (> 7 ≤28 days) n=7 |
|  | **^*^ Model 1** A**OR (95% CI)** |
| CS – ERCS | *Ref* |
| CS – TOLAC | 0.71 (0.13, 3.76) |
| **Mode of delivery 1^st^ and 2^nd^ birth** | **Infant death** (≤365 days) n=140 |
|  | **^a^ Model 1 AOR (95% CI)** |
| CS – ERCS | *Ref* |
| CS – TOLAC | 1.11 (0.76, 1.63) |

**Table**: Data are adjusted odds ratios with 95% confidence intervals. **AOR**=adjusted odds ratio**; CI**=Confidence interval, **ERCS**: Elective repeat caesarean section; **TOLAC**=Trial of labour after caesarean section;

**^*^ Model 1**: adjusted for key covariates in the second birth including maternal age, maternal country of origin, educational attainment, mother and father’s gross income, marital status, infant birthplace and infant birth weight, history of pregnancy loss and birth year (cohort n=48,711)
