# Supplementary material for: Trial of labour after caesarean section and the risk of neonatal and infant death: a nationwide cohort study
Source: BMC Pregnancy Childbirth. 2017 Feb 27;17:74. doi: 10.1186/s12884-017-1255-2 (PMC5327578; doi:10.1186/s12884-017-1255-2)
Supplement: Additional file 3: — Neonatal and infant death according to mode of delivery – sensitivity analyses with adjustment for smoking, co-morbidities and body mass index. (DOCX 16 kb) [file 12884_2017_1255_MOESM3_ESM.docx]

**Additional File 3 Neonatal and infant death according to mode of delivery – sensitivity analyses with adjustment for smoking, co-morbidities and body mass index**

| **Mode of delivery** | **Neonatal death** (≤28 days) n=95 entire cohort | | |
| --- | --- | --- | --- |
|  | **AOR (95% CI)** | | |
|  | ^*^ Model 1 (60 deaths) | **†** Model 2 (49 deaths) | **ǂ** Model 3 (23 deaths) |
| CS – ERCS | *Ref* | *Ref* | *Ref* |
| CS – TOLAC | 1.34 (0.74, 2.43) | 1.26 (0.64, 2.48) | 1.07 (0.41, 2.76) |
| **Mode of delivery** | **Early neonatal death** (≤7 days) n=86 entire cohort | | |
|  | ^*^ Model 1 (56 deaths) | **†**Model 2 (46 deaths) | **ǂ** Model 3 (23 deaths) |
| CS – ERCS | *Ref* | *Ref* | *Ref* |
| CS – TOLAC | 1.33 (0.72, 2.47) | 1.30 (0.65, 2.63) | 1.07 (0.41, 2.76) |
| **Mode of delivery** | **Late neonatal death** (> 7 days, ≤28 days) n=9 entire cohort | | |
|  | ^*^ Model 1 | **†** Model 2 | **ǂ** Model 3 |
| CS – ERCS | *Ref* | *Ref* | *Ref* |
| CS – TOLAC | *Number of deaths too few to run logistic models* | | |
| **Mode of delivery** | **Infant death** (≤365 days) n=171 entire cohort | | |
|  | ^*^ Model 1 (103 deaths) | **†**Model 2 (78 deaths) | **ǂ** Model 3 (31 deaths) |
| CS – ERCS | *Ref* | *Ref* | *Ref* |
| CS – TOLAC | 1.02 (0.65, 1.60) | 0.91 (0.54, 1.53) | 0.68 (0.31, 1.50) |

**Table**: Data are adjusted odds ratios with 95% confidence intervals. **AOR**= Adjusted odds ratio; **CI**=confidence interval, **ERCS**: Elective repeat caesarean section; **TOLAC**=Trial of labour after caesarean section**;**^*^ **Model 1**: adjusted for key covariates in the second birth including maternal age, maternal country of origin, educational attainment, mother and father’s gross income, marital status, infant birthplace and infant birth weight, history of pregnancy loss and birth year + smoking status (data available from 1991-2010 only, cohort n=50,880)

**† Model 2**: adjusted for model 1 + co-morbidities in the second delivery including hypertension, eclampsia and preeclampsia, fetal distress and gestational diabetes (data available from 1994-2010 only, cohort n=45,542)

**ǂ Model 3**: adjusted for model 2 + body mass index (BMI) (data available from 2004-2010 only, cohort n=22,672).
