# Supplementary material for: Trial of labour after caesarean section and the risk of neonatal and infant death: a nationwide cohort study
Source: BMC Pregnancy Childbirth. 2017 Feb 27;17:74. doi: 10.1186/s12884-017-1255-2 (PMC5327578; doi:10.1186/s12884-017-1255-2)
Supplement: Additional file 4: — Neonatal and infant death according to mode of delivery – sensitivity analyses without adjustment for smoking, co-morbidities and body mass index. (DOCX 16 kb) [file 12884_2017_1255_MOESM4_ESM.docx]

**Additional File 4 Neonatal and infant death according to mode of delivery – sensitivity analyses without adjustment for smoking, co-morbidities and body mass index**

| **Mode of delivery** | **Neonatal death** (≤28 days) n=95 entire cohort | | |
| --- | --- | --- | --- |
|  | **AOR (95% CI)** | | |
|  | ^*^ Model 1 (60 deaths) | **†** Model 2 (49 deaths) | **ǂ** Model 3 (23 deaths) |
| CS – ERCS | *Ref* | *Ref* | *Ref* |
| CS – TOLAC | 1.41 (0.78, 2.53) | 1.28 (0.68, 2.43) | 0.91 (0.37, 2.24) |
| **Mode of delivery** | **Early neonatal death** (≤7 days) n=86 entire cohort | | |
|  | ^*^ Model 1 (56 deaths) | **†**Model 2 (46 deaths) | **ǂ** Model 3 (23 deaths) |
| CS – ERCS | *Ref* | *Ref* | *Ref* |
| CS – TOLAC | 1.41 (0.77, 2.59) | 1.30 (0.67, 2.52) | 0.91 (0.37, 2.24) |
| **Mode of delivery** | **Late neonatal death** (> 7 days, ≤28 days) n=9 entire cohort | | |
|  | ^*^ Model 1 | **†** Model 2 | **ǂ** Model 3 |
| CS – ERCS | *Ref* | *Ref* | *Ref* |
| CS – TOLAC | *Number of deaths too few to run logistic models* | | |
| **Mode of delivery** | **Infant death** (≤365 days) n=171 entire cohort | | |
|  | ^*^ Model 1 (103 deaths) | **†**Model 2 (78 deaths) | **ǂ** Model 3 (31 deaths) |
| CS – ERCS | *Ref* | *Ref* | *Ref* |
| CS – TOLAC | 1.08 (0.69, 1.67) | 0.95 (0.58, 1.55) | 0.64 (0.30, 1.38) |

**Table**: Data are adjusted odds ratios with 95% confidence intervals. **AOR**=Adjusted odds ratio; **CI**=confidence interval, **ERCS**: Elective repeat caesarean section; **TOLAC**=Trial of labour after caesarean; **^*^ Model 1**: Cohort restricted to period for when smoking data were available (1991-2010 only, cohort n=50,880) and adjusted for key covariates in the second birth including maternal age, maternal country of origin, educational attainment, mother and father’s gross income, marital status, infant birthplace and infant birth weight, history of pregnancy loss and birth year.

**† Model 2**^:^ Cohort restricted to period for when co-morbidity data were available (1994-2010 only, cohort n=45,542) and adjusted for key covariates as in Model 1.

**ǂ Model 3:** Cohort restricted to period for when BMI data were available (2004-2010 only, cohort n=22,672) and adjusted for key covariates as in Model 1.
