# Supplementary material for: Trial of labour after caesarean section and the risk of neonatal and infant death: a nationwide cohort study
Source: BMC Pregnancy Childbirth. 2017 Feb 27;17:74. doi: 10.1186/s12884-017-1255-2 (PMC5327578; doi:10.1186/s12884-017-1255-2)
Supplement: Additional file 5: — Post-hoc analyses investigating characteristics of women with a TOLAC in Denmark according to cohort time (DOCX 12 kb) [file 12884_2017_1255_MOESM5_ESM.docx]

**Additional File 5 Post-hoc analyses investigating characteristics of women with a Successful TOLAC (VBAC) in Denmark according to cohort time**

| **Characteristics in Successful TOLAC (VBAC) group** | **Cohort time (1982-1991)** | **Cohort time (1992-2001)** | **Cohort time (2002-2010)** |
| --- | --- | --- | --- |
| VBAC Rate (%) | 7% | 8% | 7% |
| Maternal age, < 35 years | 7% | 7% | 7% |
| Maternal age > 35 years | 9% | 9% | 7% |
| Origin, Denmark | 7% | 8% | 8% |
| Other | 7% | 7% | 6% |
| Education, Primary | 7% | 7% | 6% |
| High school or more | 7% | 8% | 8% |
| Marital status, Married | 7% | 8% | 7% |
| Other | 7% | 8% | 7% |
| Gross income < 50^th^ quartile | 7% | 7% | 7% |
| Gross income > 50^th^ quartile | 7% | 8% | 8% |

**Table**: Data are presented as percentages (%) according to cohort time**. TOLAC:** Trial of Labour After Caesarean; **VBAC**: Vaginal birth after Caesarean
